# Association of Burnout With Depression and Anxiety in Critical Care Clinicians in Brazil

**DOI:** 10.1001/jamanetworkopen.2020.30898

**Published:** 2020-12-23

**Authors:** Ronald Fischer, Paulo Mattos, Cassiano Teixeira, Daniel S. Ganzerla, Regis Goulart Rosa, Fernando A. Bozza

**Affiliations:** 1 Institute D’Or for Research and Teaching, Rio de Janeiro, Brazil; 2 Victoria University of Wellington, School of Psychology, Wellington, New Zealand; 3 Instituto D’Or de Pesquisa e Ensino, Rio de Janeiro, Brazil; 4 Universidade Federal de Ciências da Saúde de Porto Alegre, Porto Alegre, Brazil; 5 Intensive Care Unit, Hospital Moinhos de Vento, Porto Alegre, Brazil; 6 Department of Critical Care, Instituto D’Or de Pesquisa e Ensino, Rio de Janeiro, Brazil; 7 Oswaldo Cruz Foundation, Rio de Janeiro, Brazil

## Abstract

**Importance:**

It is unclear whether burnout, anxiety, and depression constitute the same or different constructs. Better understanding of these constructs is important for diagnosis and treatment for intensive care unit (ICU) clinicians.

**Objective:**

To determine the associations and distinctiveness of burnout, depression, and anxiety in a sample of ICU clinicians.

**Design, Setting, and Participants:**

This cross-sectional study used baseline data from the ICU Visits Study, a cluster-randomized crossover clinical trial conducted from April 2017 to July 2018 in 36 mixed public and private nonprofit ICUs in Brazil. ICU clinicians, including day-shift physicians, nurses, nurse technicians, and physiotherapists working in an ICU at least 20 hours per week, were enrolled. Data were analyzed from December 27, 2019, to October 10, 2020.

**Main Outcomes and Measures:**

The main outcome measures were burnout, depression, and anxiety measured with the Maslach Burnout Inventory (MBI; range, 0-6, with high scores indicating more burnout) and the Hospital Depression and Anxiety Scale (HADS; range, 0-3, with higher scores indicating more depression or anxiety). Internal consistencies were satisfactory.

**Results:**

The total sample included 715 ICU clinicians (median [interquartile range] age, 34.8 [30.2-39.3] years; 520 [72.7%] women), including 96 physicians (13.4%), 159 nurses (22.2%), 358 nurse technicians (50.1%), and 102 physiotherapists (14.3%). Clinicians reported low levels of emotional exhaustion (mean [SD] score, 1.84 [1.18]), depersonalization (mean [SD] score, 0.98 [1.03]), and personal accomplishment (mean [SD] score, 5.05 [0.87]) on the MBI, and similarly low levels of depression (mean [SD] score, 0.54 [0.40]) and anxiety (mean [SD] score, 0.70 [0.45]) on the HADS. Confirmatory factor analyses consistently showed improved fit separating latent burnout dimensions from depression and anxiety. An exploratory graph analysis combining gaussian graphical model with clustering algorithms for weighted networks suggested 3 clusters, with distinct burnout, anxiety, and depression clusters. This structure was confirmed using a bootstrap with 1000 random samples, in which the 3-cluster solution emerged in 625 samples (62.5%). Both latent variable loadings and network statistics suggested 3 key indicators (ie, feeling burned out from work, worrying thoughts, and reverse-scored reporting feeling cheerful) that can be used for short screening instruments.

**Conclusions and Relevance:**

These findings suggest that burnout and clinical symptoms of depression and anxiety were empirically distinct in a large sample of ICU clinicians, highlighting the importance of screening for burnout and clinical symptoms to allow fast access to adequate support and treatment in health professionals at high risk of burnout.

## Key Points

**Question:**

Is burnout empirically distinct from depression and anxiety in intensive care unit clinicians?

**Findings:**

This cross-sectional study used baseline data from a randomized clinical trial of 715 clinicians and found that burnout was statistically distinct from anxiety and depression using both latent variable and exploratory graph analysis. Core indicators of value for inclusion in short screening instruments were identified.

**Meaning:**

These findings suggest that health professionals at high risk of stress need to be screened for both burnout and clinical symptoms, such as anxiety and depression, to provide timely and efficient treatment.

## Teaser

This cross-sectional study examines the associations of symptoms of burnout, depression, and anxiety in intensive care unit clinicians in Brazil.

## Introduction

Burnout in the medical field has attracted much attention recently, given the dramatic negative outcomes associated with burnout in medical practice and clinical outcomes. Burnout is classified as an occupational syndrome^1^ that results from chronic workplace stress that remains unresolved and that contains 3 major dimensions.^2,3^ Emotional exhaustion is the core stress dimension and entails symptoms of exhaustion and depleted emotional and physical resources; depersonalization or cynicism is the interpersonal component, including negativity, callousness, and detachment as behavioral reactions to occupational stress; and finally, lack of a feeling of personal accomplishment captures the self-evaluation of reduced efficacy and sense of accomplishment.^1,2^ Previous research has clearly demonstrated the empirical distinctiveness of these 3 components,^4,5^ with emotional exhaustion and depersonalization forming the core of burnout.^4^ Burnout has been associated with increased medical errors, increased costs for health care practitioners, and long-term adverse health outcomes.^6,7^ Professionals working in intensive care units (ICUs) are at particularly high risk of experiencing high stress and burnout, which has potentially dramatic consequences for patient safety and outcomes.^8^

However, there is currently a significant level of discussion and debate about the associations and distinctiveness of burnout with other mental health problems, including depression and anxiety.^9,10,11,12,13^ A 2018 systematic review^11^ indicated that the heterogeneity of published research does not allow a reliable examination of comorbidities, raising questions about whether it is possible to clearly distinguish burnout as an occupational syndrome from potentially underlying comorbidities. Similarly, studies in nonhealth sectors come to conflicting conclusions about the burnout-depression association. Emotional exhaustion and depression often correlate at moderate to high levels, with lower-quality studies reporting higher correlations^12^ and few high-quality studies using appropriate statistical methods to empirically test the distinctiveness of burnout from depression.^9^ The uncertainty around the possible distinctiveness raises important clinical questions for assessing the health status and providing adequate treatment options.^13^

The objective of our study is to empirically differentiate among depression, anxiety, and burnout in a representative sample of ICU clinicians, using appropriate statistical techniques and sufficient sample sizes to overcome noted problems with previous studies.

## Methods

### Study Design

We performed a subanalysis of baseline data from the ICU Visits Study,^14,15^ a cluster-randomized crossover clinical trial designed to assess the effects of a flexible ICU visiting policy on outcomes for patients, family members, and ICU clinicians. Details of trial rationale and methods have been reported previously.^14^ The ICU Visits study was conducted from April 2017 to July 2018 in 36 mixed Brazilian ICUs after approval by institutional review boards of all centers. All participating ICU clinicians provided written informed consent for study participation. The secondary analysis of data used in this cross-sectional study was covered by the original institutional review board protocol of all participating centers. Data were analyzed from December 27, 2019, to October 10, 2020.

In this secondary cross-sectional study, we assessed the overlap of burnout, depressive, and anxiety symptoms among ICU clinicians using confirmatory factor analysis (CFA) and exploratory graph analysis (EGA).^16,17,18,19^

### Population

**Figure 1.**
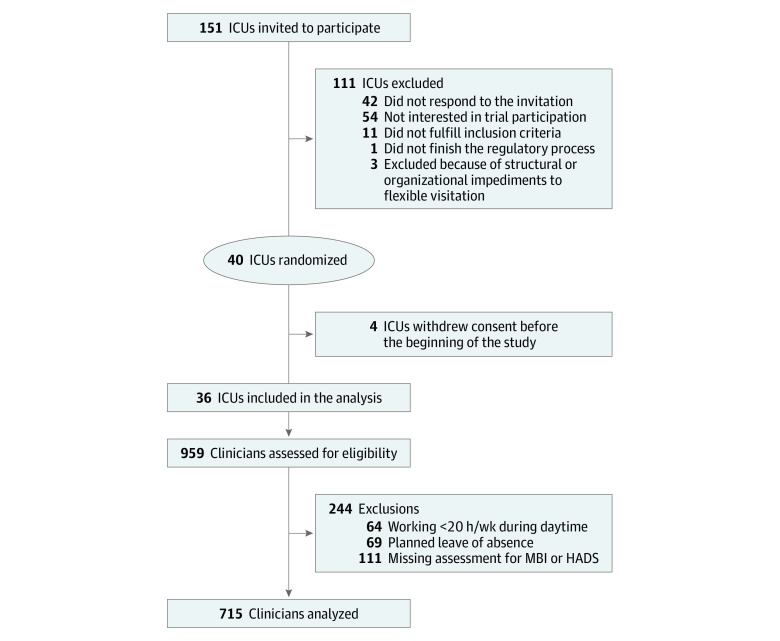
Selection Process of Participants HADS indicates Hospital Anxiety and Depression Scale; ICU, intensive care unit; and MBI, Maslach Burnout Inventory.

At the cluster level, medical-surgical ICUs from 36 hospitals in Brazil with 6 or more beds and restricted visiting hours (ie, <4.5 hours per day), including 19 public hospitals (53%) and 17 private nonprofit hospitals (47%), were enrolled. The median (interquartile range [IQR]) number of ICU beds was 13.5 (10-18) beds. At the participant level, we enrolled day-shift physicians, nurses, nurse technicians, and physiotherapists. ICU clinicians working less than 20 hours per week, planning to take a leave of absence (ie, >15 days), and those with missing values for burnout, anxiety, or depression outcomes were excluded. Details regarding exclusions are shown in Figure 1.

### Burnout, Anxiety, and Depression Symptoms

ICU clinicians were evaluated using self-administered questionnaires 2 weeks before trial interventions initiation. Burnout symptoms were assessed using the Maslach Burnout Inventory (MBI).^20^ Responses were measured on a scale from 0 (never) to 6 (every day), with higher scores indicating more burnout. A Brazilian Portuguese version was available.^21^ Anxiety and depression symptoms were measured with the Hospital Anxiety and Depression Scale (HADS),^22^ using a Brazilian version.^23^ Symptoms were measured on a scale from 0 (best) to 3 (worst), with higher scores indicating worse anxiety or depression. Both mental assessment tools were chosen based on their reported validity, objectivity, and reliability in previous research for appropriately assessing mental health symptoms in occupational contexts.^12,24,25^ In our sample, reliability estimates using ω showed good internal consistency (all estimates >0.70).^26^ Descriptive statistics and correlations at item level are available in the eTable in the Supplement.

### Statistical Analysis

We conducted a series of CFAs using the lavaan package^17^ in R statistical software version 4.0.0 (R Project for Statistical Computing). We used the diagonal-weighted least-squares estimator, given its superior performance with ordered data.^27^ Model fit was evaluated using standard fit indices, including comparative fit index, Tucker-Lewis index (values >0.9 or >0.95 are deemed acceptable in simulations using maximum likelihood estimators), root mean square error of approximation (values <0.08 or <0.06 are deemed appropriate) and standardized root mean residual (values <0.06 are deemed appropriate).^28,29^ We also report the robust χ^2^ value but do not interpret the significance level, given the well-known dependence on sample size.

We tested a series of theoretical structures to examine the statistical independence of the key constructs as measured with these 2 instruments. We first tested a 1-factor model (M1), in which all items of the MBI and HADS were forced to load on a single factor. We then tested two 2-factor models. First, we estimated a 2-factor model in which all items from the MBI and HADS loaded on their respective instrument factors, conceptually separating a burnout factor from a combined depression and anxiety latent factor (M2). A second plausible 2-factor model is one in which the emotional exhaustion and depersonalization items from the MBI and the anxiety and depression items from the HADS are forced to load on a single emotional distress and clinical symptoms factor and the personal accomplishment items from the MBI load on a efficacy factor (M3). This permits a broad test of the distinctiveness of the clinical symptoms from core components of burnout.

We then tested a series of 3 factor models that further probe the comparative distinctiveness of burnout components from clinical syndromes. First, we tested a model in which emotional exhaustion and depersonalization from the MBI and personal accomplishment from the MBI were loaded on 2 separate factors and anxiety and depression were loaded on a single factor (M4). Second, we tested a model in which the emotional exhaustion and depersonalization items from the MBI and the depression items from the HADS were loaded on a single factor (testing the depression association of burnout), personal accomplishment was loaded on a second factor, and anxiety was loaded on a third factor (M5). Third, we tested the anxiety association of emotional exhaustion and depersonalization by forcing the core components of the MBI and anxiety items from the HADS to load on a single factor, depression items and personal accomplishment items were loaded on their separate factors (M6). Fourth, we tested a model in which we forced all burnout items to load on a single burnout factor and allowed separate anxiety and depression factors (M7).

An additional model included 2 burnout factors (separating personal accomplishment from a combined emotional exhaustion and depersonalization factor as burnout core^3,4^) and separate anxiety and depression factors (M8). A second 4-factor model separated the 3 MBI factors from a combined HADS anxiety and depression factor (M9).

Finally, a full 5-factor model separated all the theoretically estimated dimensions, 3 different burnout dimensions and separate anxiety and depression factors (M10).

We also tested whether the best fitting model differed for the different professions. We ran a multigroup invariance test, with increasing restrictive equality assumptions across models.^30^ We first tested a configural model with no equality constraints across groups, in the next step constrained the factor loadings, and finally constrained the item intercepts to be equal.

We then used a network analysis approach, which is ideally suited to uncover possible associations between symptoms reported in psychological inventories, especially in the context of comorbidities.^31,32,33^ To overcome problems with latent confounding,^34^ we tested the distinctiveness of network clusters with bootstrapped EGA, using extended bayesian information criterion (EBIC) graphical least absolute shrinkage and selection operator (GLASSO) estimation.^35^ The GLASSO^36^ is a regression-based approach that shrinks coefficients to obtain a network that faithfully represents the network while also reducing near-zero correlations (correlations are represented as edges between nodes in network systems) to exact zero.^32^ The EBIC GLASSO method has been shown to work particularly well in retrieving a true network structure.^37^ To identify network communities, we used a walktrap algorithm,^38^ which has been shown to be superior to standard methods for identifying optimal number of clusters.^19^ To overcome potential instabilities and accuracy problems in sample specific solutions, we bootstrapped the EGA results using 1000 samples.^32^

## Results

### Participants

A total of 959 clinicians were assessed for eligibility (Figure 1). After excluding possible participants not meeting the inclusion criteria, 715 ICU clinicians were included in the present analysis, including 96 physicians (13.4%), 159 nurses (22.2%), 358 nurse technicians (50.1%), and 102 physiotherapists (14.3%). Table 1 summarizes the characteristics of the study population. Median (IQR) age was 34.8 (30.2-39.3) years, and 520 (72.7%) were women. The median (IQR) number of years of experience in ICU work was 5.2 (2.1-10.0) years, and median (IQR) working hours per week was 40 (36-60) hours.

**Table 1.** Participant Demographic Characteristics

Characteristic	No. (%) (N = 715)
Age, median (IQR), y	34.8 (30.2-39.3)
Sex	
Men	195 (27.3)
Women	520 (72.7)
Has children	384 (53.8)
Marital status	
Married or cohabitating	371 (52.3)
Divorced	52 (7.3)
Single	280 (39.4)
Widowed	7 (1.0)
Occupation^a^	
Physician	96 (13.4)
Nurse	159 (22.2)
Nurse technician	358 (50.1)
Physiotherapist	102 (14.3)
ICU experience, median (IQR), y	5.2 (2.1-10)
Workload, median (IQR), h/wk	40 (36-60)
Patients per clinician, median (IQR), No.	
Physician	10 (6.5-10)
Nurse	8 (5-10)
Nurse technician	2 (2-2)
Physiotherapist	10 (8-10)
Clinical status	
Anxiety symptoms^b^	134 (18.7)
Depression symptoms^b^	80 (11.2)
Emotional exhaustion^c^	125 (17.5)
Depersonalization^c^	120 (16.8)
Personal accomplishment^c^	107 (15.0)

Abbreviations: ICU, intensive care unit; IQR, interquartile range.

^a^ In Brazil, bedside nursing care is often delivered by nurse technicians under supervision of a nurse.

^b^ Defined as a score greater than 7 for that aspect on the Hospital Anxiety and Depression Scale.

^c^ Defined using responses to the Maslach Burnout Inventory for each aspect. Emotional exhaustion was considered a score of greater than 13; depersonalization, greater than 10; and personal accomplishment, less than 33.

On the MBI, participants reported overall low levels of emotional exhaustion (mean [SD] score, 1.84 [1.18]) and depersonalization (mean [SD] score, 0.98 [1.03]) and high levels of personal accomplishment (mean [SD] score, 5.05 [0.87]). Similarly, on the HADS, participants reported low levels depression (mean [SD] score, 0.54 [0.40]) and anxiety (mean [SD] score, 0.70 [0.45]).

### CFA

The CFA analyses showed that across all possible comparisons, a solution combining the core burnout dimensions of emotional exhaustion and depersonalization with either anxiety or depression or both combined always fit worse compared with a model that separated burnout from anxiety and depression (Table 2). The best fit overall was found for M10, the theoretically estimated 5-factor model separating the 3 burnout dimensions and the 2 clinical symptoms. Table 2 shows the fit indices for the individual models, and Table 3 shows the factor loadings and latent variable intercorrelations. The correlations of the latent variables supported that the 2 core burnout dimensions correlated more strongly with each other than with either depression or anxiety (change in *r* = 0.02 to 0.13).

**Table 2.** Model Fit Parameters From the Confirmatory Factor Analysis

Model	Model specification	Robust χ^2^	*df*	CFI	TLI	RMSEA (95% CI)	SRMR	Change, χ^2^
M1: 1 Factor	Single factor	1831.21	594	0.928	0.924	0.055 (0.052-0.058)	0.077	NA
M2: 2 Factors	MBI and HADS	1451.28	593	0.955	0.952	0.044 (0.041-0.046)	0.067	379.93
M3: 2 factors	EE + DP + HADS, and PA	1583.33	593	0.95	0.946	0.046 (0.043-0.049)	0.065	247.88
M4: 3 factors	EE + DP, PA, and HADS	1108.08	591	0.982	0.981	0.028 (0.024-0.031)	0.051	343.2
M5: 3 factors	EE + DP + Dep, PA, and Anx	1496.97	591	0.956	0.954	0.043 (0.040-0.046)	0.062	86.36
M6: 3 factors	EE + DP + Anx, PA, and Dep	1463.02	591	0.958	0.955	0.042 (0.039-0.045)	0.062	120.31
M7: 3 factors	MBI, Anx, and Dep	1435.86	591	0.956	0.953	0.043 (0.040-0.046)	0.066	395.35
M8: 4 factors	EE + DP, PA, Anx, and Dep	1076.05	588	0.984	0.983	0.026 (0.022-0.029)	0.05	32.03
M9: 4 factors	EE, DP, PA, and HADS	1025.27	588	0.987	0.987	0.023 (0.019-0.027)	0.048	82.81
M10: 5 factors	EE, DP, PA, Anx, Dep	991.52	584	0.99	0.989	0.021 (0.017-0.025)	0.047	33.75

Abbreviations: Anx, anxiety; Dep, depression; DP, depersonalization; CFI, comparative fit index; EE, emotional exhaustion; HADS, Hospital Anxiety and Depression Scale; MBI, Maslach Burnout Inventory; NA, not applicable; PA, personal accomplishment; RMSEA, root mean square error of approximation; SRMR, standardized root mean residual; TLI, Tucker-Lewis index.

**Table 3.** Fully Standardized Factor Loadings, Latent Factor Correlations, and Network Centrality Parameters

Item	Standardized factor loadings λ					Network degree centrality, EBIC GLASSO estimation
	Emotional exhaustion	Depersonalization	Personal accomplishment	Anxiety	Depression	
Emotionally drained from work	0.66	NA	NA	NA	NA	0.93
Feel used up at the end of the workday	0.53	NA	NA	NA	NA	0.76
Feel fatigued when getting up	0.69	NA	NA	NA	NA	1.08
Working with people puts too much stress	0.64	NA	NA	NA	NA	0.95
Feel burned out from work	0.77	NA	NA	NA	NA	1.29
Feel frustrated by job	0.63	NA	NA	NA	NA	0.93
Feel working too hard on the job	0.65	NA	NA	NA	NA	0.90
Working with patients is a drain	0.64	NA	NA	NA	NA	1.02
Feel like at the end of the rope	0.69	NA	NA	NA	NA	1.02
Treat patients as impersonal objects	NA	0.49	NA	NA	NA	0.60
Become more callous toward people	NA	0.70	NA	NA	NA	0.98
Worry that job is hardening emotionally	NA	0.71	NA	NA	NA	1.04
Do not really care what happens to patients	NA	0.25	NA	NA	NA	0.34
Felt patients blame them for problems	NA	0.57	NA	NA	NA	0.59
Can easily understand patients' feelings	NA	NA	0.24	NA	NA	0.45
Deal effectively with the patients' problems	NA	NA	0.35	NA	NA	0.77
Feel positively influencing people's lives	NA	NA	0.46	NA	NA	0.85
Feel very energetic	NA	NA	0.45	NA	NA	0.58
Can easily create a relaxed atmosphere	NA	NA	0.56	NA	NA	0.83
Feel exhilarated after working with patients	NA	NA	0.71	NA	NA	1.04
Having accomplished worthwhile things in job	NA	NA	0.69	NA	NA	0.84
Deal with emotional problems calmly	NA	NA	0.54	NA	NA	0.80
Tense or wound up	NA	NA	NA	0.66	NA	0.98
Frightened feeling as if something awful is about to happen	NA	NA	NA	0.55	NA	0.98
Worrying thoughts go through mind	NA	NA	NA	0.69	NA	1.10
Sit at ease and feel relaxed	NA	NA	NA	0.53	NA	0.84
Frightened feeling like butterflies in stomach	NA	NA	NA	0.51	NA	0.86
Restless and have to be on the move	NA	NA	NA	0.52	NA	0.81
Sudden feelings of panic	NA	NA	NA	0.49	NA	0.77
Enjoy the things I used to enjoy	NA	NA	NA	NA	0.61	0.96
Laugh and see the funny side of things	NA	NA	NA	NA	0.54	0.84
Cheerful	NA	NA	NA	NA	0.60	0.97
Slowed down	NA	NA	NA	NA	0.40	0.54
Lost interest in my appearance	NA	NA	NA	NA	0.56	0.86
Look forward with enjoyment to things	NA	NA	NA	NA	0.60	0.90
Enjoy a good book or television program	NA	NA	NA	NA	0.45	0.70
Emotional exhaustion	0.87^a^	NA	NA	NA	NA	NA
Depersonalization	0.68^a^	0.78^a^	NA	NA	NA	NA
Personal accomplishment	–0.41^a^	–0.39^a^	0.75^a^	NA	NA	NA
Anxiety	0.62^a^	0.55^a^	–0.40^a^	0.77^a^	NA	NA
Depression	0.59^a^	0.55^a^	–0.51^a^	0.85^a^	0.74^a^	NA

Abbreviations: EBIC, extended bayesian information criterion; GLASSO, graphical least absolute shrinkage and selection operator; NA, not applicable.

^a^ Latent variable correlations (latent variable variance is set to 1).

### EGA

**Figure 2.**
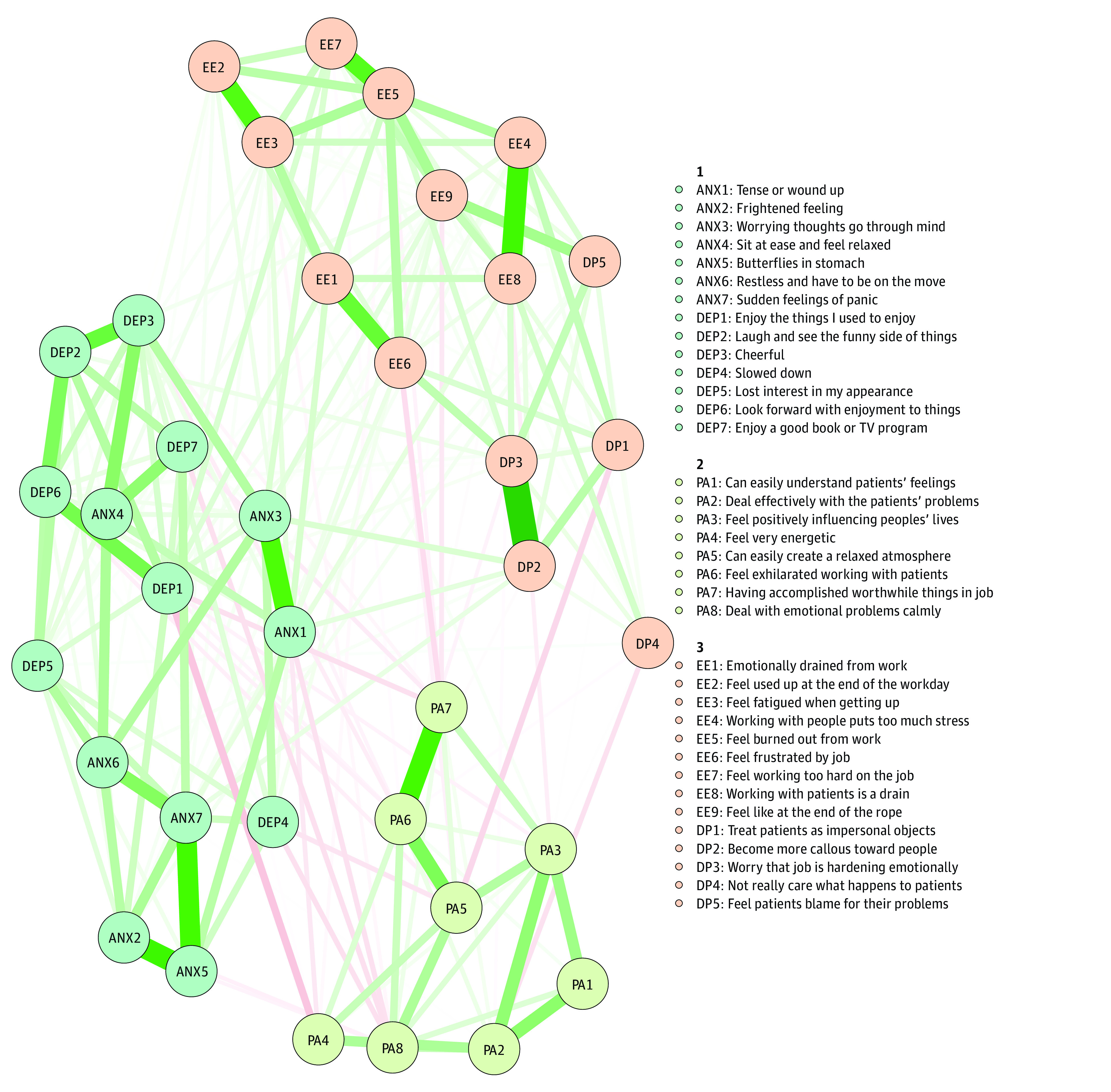
Community Structure Estimated Using Exploratory Graph Analysis Nodes with different colors indicate community membership; green lines, positive associations; pink lines, negative associations. The legend identifies variables associated with each community, including anxiety (ANX), depression (DEP), personal accomplishment (PA), emotional exhaustion (EE), and depersonalization (DP).

An EGA showed 3 distinct clusters within our network. Figure 2 shows the cluster membership. Cluster 1 combined anxiety and depression scales from the HADS; cluster 2 featured the personal accomplishment items from the MBI, and cluster 3 combined the emotional exhaustion and depersonalization MBI items. This 3-cluster solution emerged in 625 of 1000 bootstrap samples (62.5%), while a 4-cluster solution further separating emotional exhaustion and depersonalization items emerged in 281 bootstrap samples (28.1%). A solution statistically assigning burnout and depression items to the same cluster never emerged in any of the 1000 bootstrap solutions. Therefore, a 3-cluster solution is most consistent with the data, clearly differentiating burnout from depression and anxiety. This cluster solution is identical to M4. Some other latent variable CFA models showed equally or better model fit (eg, M8, M9, M10), suggesting that finer distinctions between the core burnout dimensions and anxiety and depression could improve model fit. What bootstrapped EGA provides is an additional estimate of the most parsimonious and stable clustering solution, therefore taking into consideration both model fit and parsimony based on 1000 bootstrap samples.

### Core Indicators

Both factor loadings and network centrality parameters allow the identification of salient indicators within the burnout-depression-anxiety network (Table 3). Burnout item 8 (I feel burned out from my work), anxiety item 5 (worrying thoughts go through my mind), and depression item 6 (I feel cheerful [reverse scored]) are strongly connected in the overall network, suggesting their suitability as brief markers to differentiate burnout from other mental health problems. These network centrality indicators converge with the standardized factor loadings from the best fitting model. In our sample, the network centrality indicators correlated 0.86 with the *R^2^* values (capturing the extent to which latent variables explain variability in the endorsement of the individual items) and 0.87 with the standardized factor loadings from M10, the 5-factor CFA model. Therefore, the analyses converge and confirm recently identified core items for short screening instruments.^39^

## Discussion

This cross-sectional study found that burnout and depression are correlated but empirically distinct latent factors and from distinct networks and that emotional exhaustion and depersonalization form the core of the burnout construct, highlighting that the distinctions within the burnout construct might be of clinical importance. Additionally, we identified 3 central items that were core symptoms of burnout, depression, and anxiety, which could be tracked in large populations using short scales.^39^

The differentiation of burnout from related mental health problems is clinically important because it may be less stigmatizing to classify a physician’s distress as burnout, but it “may prevent or delay appropriate treatment of MDD [major depressive disorder], a serious and sometimes life-threatening mental disorder,” as suggested by Oquendo et al,^13^ leading to misdiagnosis of symptoms and inappropriate and possibly harmful interventions. Therefore, identifying whether these symptoms are overlapping or distinct has important consequences for efficient diagnosis and delivery of adequate treatment options, which impact both the well-being and effectiveness of clinicians as well as health outcomes of patients in critical care units, especially in the context of the increased demands on ICUs associated with the coronavirus disease 2019 pandemic.

The clinical implications of our findings are noteworthy, because previous research has found inconsistent associations between burnout and depression. Using state-of-the-art latent variable and EGAs, which are ideally suited to identify associations with potential comorbidity, burnout and depression are empirically distinct in this high-risk population of clinicians working in ICUs. Burnout itself appears to have 2 distinct components, pointing to a greater need to differentiate clinical profiles of burnout. The combined emotional exhaustion and depersonalization component is more central within the larger network, pointing to the greater clinical relevance.^8^ Furthermore, by examining the network parameters of the individual indicators, we identified core behavioral indicators that are central within the burnout and depression networks. These core indicators can be used to rapidly and easily screen both depression and burnout in health care workers, allowing fast access to adequate support and treatment, which is of utmost importance in the current pandemic.

### Limitations

This study has several limitations. First, the randomized clinical trial from which we extracted our data was not primarily designed to assess the associations between burnout, depression, and anxiety symptoms among ICU clinicians. Therefore, our results should be considered exploratory. Second, although the study recruited a relatively large sample of ICUs and ICU clinicians, the conclusions might be limited to the middle-income sociocultural context where the study was conducted. Thus, distinct results are possible in different sociocultural settings. All data were collected using self reports, and this strategy may have resulted in a higher proportion of missing values for MBI and HADS questionnaires than a face-to-face interview would have. Independent assessment by trained clinicians could be included in future studies. We used both theory-driven and exploratory statistical methods to empirically evaluate the overlap between the constructs. The theoretically derived 5-factor structure provided the best fit, but the exploratory EGA approach suggested that a 3-cluster solution is more parsimonious, suggesting that different choices of statistical techniques and cutoff criteria would lead to different conclusions. These findings need replication in new samples using different instruments and a wider variety of statistical techniques.

## Conclusions

These findings suggest that burnout was empirically distinct from depression and anxiety in population of ICU clinicians who were at high risk of job stress and burnout. Practitioners should screen for burnout as a work-related stress syndrome and for clinical syndromes, such as depression and anxiety, to provide appropriate diagnosis and offer appropriate treatment. Our analysis offers options for measuring core constructs for screening purposes.

## Notes

Supplement. **eTable.** Descriptive Information, Including Means, SDs, and Item Intercorrelations
