# Supplementary material for: Association of Burnout With Depression and Anxiety in Critical Care Clinicians in Brazil
Source: JAMA Netw Open. 2020 Dec 23;3(12):e2030898. doi: 10.1001/jamanetworkopen.2020.30898 (PMC7758805; doi:10.1001/jamanetworkopen.2020.30898)
Supplement: Supplement. — eTable. Descriptive Information, Including Means, SDs, and Item Intercorrelations [file jamanetwopen-e2030898-s001.pdf]

Supplemental Online Content

Fischer R, Mattos P, Teixeira C, Ganzerla DS, Rosa RG, Bozza FA. Association of burnout with depression and anxiety in critical care clinicians in Brazil. *JAMA Netw Open*. 2020;3(12):e2030898. doi:10.1001/jamanetworkopen.2020.30898

eTable. Descriptive Information, Including Means, SDs, and Item Intercorrelations

This supplemental material has been provided by the authors to give readers additional information about their work.

© 2020 Fischer R et al. *JAMA Network Open.*

eTable. Descriptive Information, Including Means, SDs, and Item Intercorrelations

Means, standard deviations, and correlations with confidence intervals																																					
Variable	*M*	*SD*	1	2	3	4	5	6	7	8	9	10	11	12	13	14	15	16	17	18	19	20	21	22	23	24	25	26	27	28	29	30	31	32	33	34	35
1. EE1	1.73	1.63																																			
2. EE2	3.45	1.67	.40**																																		
			[.34, .46]																																		
3. EE3	1.88	1.78	.48**	.56**																																	
			[.42, .53]	[.51, .60]																																	
4. EE4	1.16	1.57	.39**	.32**	.41**																																
			[.33, .45]	[.26, .38]	[.35, .47]																																
5. EE5	2.03	1.88	.48**	.48**	.54**	.49**																															
			[.43, .53]	[.42, .53]	[.49, .59]	[.43, .54]																															
6. EE6	1.16	1.51	.53**	.26**	.42**	.38**	.49**																														
			[.48, .58]	[.19, .32]	[.36, .47]	[.32, .43]	[.44, .54]																														
7. EE7	2.57	2.01	.38**	.42**	.46**	.38**	.60**	.37**																													
			[.32, .44]	[.36, .48]	[.41, .51]	[.32, .44]	[.56, .64]	[.31, .43]																													
8. EE8	1.22	1.51	.41**	.32**	.34**	.57**	.45**	.38**	.40**																												
			[.35, .46]	[.25, .38]	[.28, .40]	[.53, .62]	[.39, .50]	[.32, .43]	[.34, .46]																												
9. EE9	1.35	1.64	.38**	.30**	.38**	.37**	.50**	.38**	.44**	.41**																											
			[.32, .44]	[.24, .36]	[.32, .43]	[.31, .43]	[.45, .55]	[.32, .44]	[.38, .50]	[.35, .46]																											
10. DP1	0.57	1.2	.26**	.14**	.24**	.33**	.31**	.30**	.21**	.28**	.20**																										
			[.19, .32]	[.07, .21]	[.17, .30]	[.27, .39]	[.25, .37]	[.23, .36]	[.15, .28]	[.21, .34]	[.14, .27]																										
11. DP2	1.53	1.9	.27**	.14**	.26**	.34**	.33**	.29**	.29**	.34**	.30**	.34**																									
			[.20, .33]	[.07, .21]	[.19, .32]	[.28, .40]	[.27, .39]	[.22, .35]	[.23, .35]	[.28, .40]	[.24, .36]	[.28, .40]																									
12. DP3	1.47	1.84	.31**	.19**	.31**	.34**	.40**	.39**	.34**	.37**	.33**	.32**	.62**																								
			[.25, .37]	[.12, .26]	[.25, .37]	[.28, .40]	[.34, .46]	[.33, .45]	[.27, .40]	[.31, .43]	[.26, .39]	[.25, .38]	[.58, .66]																								

© 2020 Fischer R et al. *JAMA Network Open.*

13. DP4	0.31	1.04	.12**	.08*	.08*	.20**	.13**	.12**	.16**	.23**	.17**	.15**	.19**	.20**																							
			[.05, .19]	[.01, .15]	[.01, .15]	[.13, .26]	[.07, .20]	[.05, .19]	[.10, .23]	[.16, .29]	[.11, .24]	[.09, .22]	[.12, .26]	[.13, .27]																							
14. DP5	0.98	1.56	.25**	.17**	.26**	.30**	.30**	.26**	.29**	.27**	.36**	.23**	.30**	.33**	.15**																						
			[.19, .32]	[.10, .23]	[.19, .32]	[.24, .36]	[.24, .36]	[.19, .32]	[.22, .35]	[.21, .34]	[.30, .42]	[.17, .30]	[.24, .36]	[.27, .39]	[.08, .22]																						
15. PA1	5.46	1.19	0	0.01	-0.01	-0.06	0	-0.01	-0.02	-.10**	-0.06	-0.04	-0.03	-.10**	-.07*	-0.04																					
			[-.07, .07]	[-.06, .07]	[-.08, .06]	[-.13, .00]	[-.07, .07]	[-.07, .06]	[-.09, .05]	[-.17, -.03]	[-.13, .00]	[-.11, .03]	[-.10, .04]	[-.16, -.03]	[-.14, -.00]	[-.11, .02]																					
16. PA2	5.1	1.5	-.08*	-0.03	-0.04	-.07*	-0.06	-.07*	-.07*	-.15**	-.10**	-.09**	-.07*	-.10**	-.14**	-.08*	.33**																				
			[-.15, -.01]	[-.10, .04]	[-.11, .03]	[-.14, -.00]	[-.12, .01]	[-.14, -.00]	[-.14, -.00]	[-.22, -.08]	[-.17, -.03]	[-.16, -.02]	[-.14, -.00]	[-.17, -.03]	[-.21, -.08]	[-.15, -.02]	[.27, .39]																				
17. PA3	5.22	1.34	-.12**	0.01	-.12**	-.15**	-.11**	-.14**	-0.05	-.18**	-.11**	-.14**	-.08*	-.15**	-.12**	-0.05	.31**	.35**																			
			[-.19, -.05]	[-.05, .08]	[-.18, -.05]	[-.22, -.08]	[-.18, -.04]	[-.20, -.07]	[-.11, .02]	[-.24, -.11]	[-.18, -.04]	[-.20, -.07]	[-.15, -.01]	[-.22, -.08]	[-.19, -.05]	[-.12, .02]	[.25, .37]	[.29, .41]																			
18. PA4	4.39	1.82	-.11**	-.08*	-.16**	-.10**	-.19**	-.11**	-.16**	-.09*	-.16**	-0.05	-.12**	-.08*	0	-0.04	.14**	.28**	.21**																		
			[-.17, -.04]	[-.15, -.01]	[-.22, -.09]	[-.16, -.03]	[-.25, -.12]	[-.18, -.04]	[-.22, -.09]	[-.15, -.02]	[-.23, -.10]	[-.12, .02]	[-.18, -.05]	[-.15, -.01]	[-.07, .07]	[-.11, .03]	[.07, .21]	[.21, .34]	[.14, .28]																		
19. PA5	5.17	1.39	-.17**	-0.05	-.12**	-.16**	-.18**	-.21**	-.12**	-.11**	-.18**	-.20**	-.08*	-.18**	-0.03	-.11**	.16**	.19**	.31**	.27**																	
			[-.24, -.10]	[-.12, .02]	[-.18, -.05]	[-.22, -.09]	[-.24, -.11]	[-.27, -.14]	[-.18, -.05]	[-.18, -.04]	[-.24, -.11]	[-.27, -.13]	[-.15, -.01]	[-.24, -.11]	[-.10, .04]	[-.17, -.04]	[.09, .22]	[.12, .25]	[.24, .37]	[.20, .33]																	
20. PA6	5.06	1.38	-.18**	-0.06	-.15**	-.18**	-.23**	-.21**	-.22**	-.21**	-.22**	-.17**	-.20**	-.22**	-.13**	-.15**	.18**	.24**	.29**	.27**	.42**																
			[-.25, -.12]	[-.13, .01]	[-.21, -.08]	[-.24, -.11]	[-.30, -.17]	[-.27, -.14]	[-.28, -.15]	[-.27, -.14]	[-.28, -.15]	[-.24, -.10]	[-.27, -.14]	[-.28, -.15]	[-.20, -.06]	[-.21, -.08]	[.11, .24]	[.18, .31]	[.22, .35]	[.21, .34]	[.36, .48]																
21. PA7	5.11	1.46	-.22**	-.12**	-.21**	-.18**	-.25**	-.26**	-.18**	-.21**	-.24**	-.16**	-.18**	-.19**	-.08*	-.14**	.16**	.22**	.28**	.25**	.31**	.50**															
			[-.28, -.15]	[-.18, -.05]	[-.27, -.14]	[-.25, -.12]	[-.32, -.19]	[-.32, -.19]	[-.24, -.11]	[-.27, -.14]	[-.31, -.18]	[-.23, -.09]	[-.24, -.11]	[-.25, -.12]	[-.15, -.01]	[-.20, -.07]	[.09, .22]	[.15, .28]	[.22, .35]	[.18, .31]	[.25, .37]	[.45, .55]															
22. PA8	4.91	1.58	-.18**	-.09*	-.20**	-.17**	-.15**	-.16**	-.13**	-.17**	-.19**	-.13**	-.12**	-.17**	-0.03	-.09**	.21**	.27**	.27**	.19**	.32**	.31**	.26**														

© 2020 Fischer R et al. *JAMA Network Open.*

			[-.25, -.11]	[-.15, -.02]	[-.26, -.13]	[-.23, -.10]	[-.21, -.08]	[-.23, -.10]	[-.19, -.06]	[-.24, -.10]	[-.26, -.12]	[-.20, -.07]	[-.18, -.05]	[-.23, -.10]	[-.10, .04]	[-.16, -.02]	[.15, .28]	[.20, .33]	[.20, .33]	[.13, .26]	[.26, .38]	[.25, .37]	[.19, .32]														
23. ANX1	1.02	0.64	.31**	.27**	.31**	.26**	.31**	.29**	.28**	.27**	.33**	.18**	.30**	.24**	.08*	.22**	-0.03	-0.04	-.08*	-.18**	-.15**	-.19**	-.20**	-.22**													
			[.25, .37]	[.21, .33]	[.25, .38]	[.19, .32]	[.25, .37]	[.22, .35]	[.21, .34]	[.20, .33]	[.27, .39]	[.11, .24]	[.24, .36]	[.18, .31]	[.01, .15]	[.16, .29]	[-.09, .04]	[-.11, .03]	[-.14, -.01]	[-.25, -.11]	[-.22, -.08]	[-.26, -.13]	[-.27, -.13]	[-.29, -.16]													
24. ANX2	0.61	0.8	.19**	.20**	.24**	.18**	.19**	.19**	.14**	.20**	.27**	.08*	.22**	.23**	.09**	.20**	-0.05	-.08*	-.08*	-.11**	-.08*	-.17**	-.13**	-.21**	.28**												
			[.12, .25]	[.13, .26]	[.18, .31]	[.11, .25]	[.12, .25]	[.12, .25]	[.07, .20]	[.13, .26]	[.21, .33]	[.01, .15]	[.15, .28]	[.16, .29]	[.02, .16]	[.14, .27]	[-.12, .01]	[-.15, -.02]	[-.15, -.01]	[-.18, -.04]	[-.15, -.01]	[-.24, -.10]	[-.20, -.06]	[-.28, -.14]	[.22, .34]												
25. ANX3	1.12	0.82	.27**	.21**	.30**	.28**	.33**	.28**	.33**	.27**	.35**	.15**	.33**	.28**	.08*	.20**	-0.05	-0.07	-.10**	-.13**	-.17**	-.23**	-.14**	-.19**	.50**	.31**											
			[.20, .33]	[.15, .28]	[.24, .37]	[.22, .35]	[.26, .39]	[.22, .34]	[.27, .39]	[.21, .33]	[.29, .41]	[.08, .22]	[.27, .39]	[.22, .34]	[.01, .14]	[.13, .26]	[-.12, .02]	[-.13, .00]	[-.16, -.03]	[-.20, -.07]	[-.23, -.10]	[-.30, -.17]	[-.21, -.07]	[-.25, -.12]	[.44, .55]	[.25, .37]											
26. ANX4	0.98	0.8	.22**	.24**	.28**	.20**	.20**	.19**	.24**	.16**	.25**	0.06	.18**	.17**	.09**	.12**	0.01	-.09*	-.08*	-.12**	-0.07	-.13**	-.14**	-.15**	.33**	.26**	.36**										
			[.15, .28]	[.18, .30]	[.21, .34]	[.13, .26]	[.14, .27]	[.12, .25]	[.17, .30]	[.09, .22]	[.19, .32]	[-.01, .13]	[.12, .25]	[.10, .24]	[.02, .16]	[.05, .19]	[-.06, .08]	[-.16, -.02]	[-.15, -.01]	[-.19, -.05]	[-.14, .00]	[-.19, -.06]	[-.21, -.07]	[-.21, -.08]	[.26, .39]	[.20, .32]	[.30, .42]										
27. ANX5	0.5	0.58	.17**	.16**	.20**	.17**	.17**	.18**	.13**	.19**	.20**	.11**	.20**	.20**	0.04	.14**	-0.05	-.11**	-.10**	-.12**	-.12**	-.10**	-.10**	-.20**	.32**	.50**	.30**	.24**									
			[.10, .23]	[.09, .23]	[.14, .27]	[.10, .23]	[.10, .24]	[.11, .24]	[.07, .20]	[.12, .25]	[.13, .27]	[.04, .17]	[.14, .27]	[.13, .27]	[-.03, .11]	[.08, .21]	[-.12, .02]	[-.17, -.04]	[-.17, -.03]	[-.19, -.05]	[-.19, -.05]	[-.17, -.04]	[-.17, -.04]	[-.27, -.13]	[.26, .38]	[.45, .55]	[.24, .36]	[.17, .30]									
28. ANX6	0.55	0.78	.17**	.21**	.21**	.20**	.19**	.14**	.19**	.21**	.24**	.10**	.25**	.22**	0.03	.16**	-.08*	-.11**	-.09*	-.10**	-.16**	-.18**	-.10**	-.15**	.29**	.37**	.36**	.33**	.31**								
			[.10, .23]	[.15, .28]	[.14, .27]	[.13, .26]	[.12, .25]	[.08, .21]	[.12, .26]	[.14, .28]	[.17, .30]	[.03, .17]	[.18, .31]	[.16, .29]	[-.04, .10]	[.09, .22]	[-.15, -.01]	[-.18, -.05]	[-.16, -.02]	[-.17, -.03]	[-.23, -.09]	[-.24, -.11]	[-.17, -.03]	[-.22, -.09]	[.23, .35]	[.31, .43]	[.30, .42]	[.27, .39]	[.24, .37]								
29. ANX7	0.16	0.43	.18**	.16**	.22**	.22**	.21**	.19**	.17**	.24**	.22**	.15**	.24**	.17**	0.05	.14**	0	-0.03	-0.02	-.07*	-0.05	-.11**	-.11**	-.12**	.27**	.38**	.25**	.23**	.40**	.33**							
			[.11, .24]	[.10, .23]	[.15, .28]	[.15, .28]	[.14, .28]	[.12, .25]	[.10, .23]	[.18, .31]	[.16, .29]	[.08, .21]	[.17, .30]	[.10, .23]	[-.01, .12]	[.08, .21]	[-.07, .07]	[-.09, .04]	[-.09, .05]	[-.14, -.01]	[-.12, .01]	[-.18, -.04]	[-.18, -.04]	[-.19, -.06]	[.20, .33]	[.32, .44]	[.18, .31]	[.16, .29]	[.34, .45]	[.27, .39]							
30. DEP1	0.51	0.6	.30**	.21**	.30**	.22**	.30**	.29**	.30**	.22**	.30**	.19**	.24**	.26**	.13**	.20**	-0.03	-.10**	-.18**	-.23**	-.18**	-.25**	-.26**	-.18**	.25**	.30**	.27**	.27**	.20**	.18**	.17**						
			[.24, .36]	[.14, .27]	[.23, .36]	[.16, .29]	[.24, .36]	[.23, .35]	[.24, .36]	[.16, .29]	[.23, .36]	[.12, .25]	[.17, .30]	[.20, .33]	[.06, .20]	[.13, .26]	[-.10, .04]	[-.17, -.03]	[-.25, -.12]	[-.29, -.16]	[-.25, -.12]	[-.31, -.18]	[-.32, -.19]	[-.25, -.11]	[.18, .31]	[.23, .36]	[.21, .33]	[.20, .33]	[.13, .26]	[.11, .24]	[.11, .24]						
31. DEP2	0.3	0.55	.16**	.14**	.21**	.18**	.19**	.21**	.12**	.19**	.26**	.13**	.17**	.25**	.09**	.16**	0	-0.06	-.13**	-.08*	-.15**	-.21**	-.18**	-.17**	.24**	.28**	.29**	.29**	.21**	.25**	.22**	.35**					

© 2020 Fischer R et al. *JAMA Network Open.*

			[.09, .23]	[.08, .21]	[.14, .27]	[.11, .25]	[.13, .26]	[.15, .28]	[.05, .19]	[.13, .26]	[.20, .33]	[.07, .20]	[.10, .24]	[.19, .32]	[.02, .16]	[.09, .22]	[-.06, .07]	[-.12, .01]	[-.19, -.06]	[-.15, -.01]	[-.21, -.08]	[-.27, -.14]	[-.25, -.11]	[-.24, -.11]	[.17, .30]	[.22, .34]	[.23, .35]	[.22, .35]	[.14, .27]	[.18, .31]	[.15, .29]	[.28, .40]					
32. DEP3	0.66	0.63	.19**	.11**	.18**	.21**	.22**	.20**	.20**	.19**	.28**	.16**	.22**	.22**	.08*	.14**	-0.02	-.12**	-.17**	-.17**	-.18**	-.23**	-.16**	-.17**	.32**	.25**	.39**	.41**	.20**	.26**	.22**	.34**	.41**				
			[.13, .26]	[.04, .18]	[.11, .24]	[.14, .27]	[.15, .28]	[.14, .27]	[.13, .26]	[.13, .26]	[.22, .35]	[.09, .22]	[.15, .28]	[.15, .28]	[.01, .15]	[.07, .20]	[-.08, .05]	[-.18, -.05]	[-.23, -.10]	[-.24, -.10]	[-.25, -.11]	[-.30, -.17]	[-.22, -.09]	[-.24, -.11]	[.26, .38]	[.18, .31]	[.33, .44]	[.35, .46]	[.14, .27]	[.20, .32]	[.15, .28]	[.28, .40]	[.35, .47]				
33. DEP4	0.9	0.63	.13**	.12**	.22**	.17**	.17**	.19**	.19**	.18**	.21**	.10**	.18**	.15**	0.03	.12**	-0.03	-0.05	-0.07	-.12**	-.17**	-.12**	-0.07	-.18**	.25**	.21**	.30**	.13**	.25**	.21**	.21**	.18**	.11**	.23**			
			[.06, .20]	[.05, .18]	[.15, .28]	[.10, .23]	[.11, .24]	[.12, .25]	[.13, .26]	[.12, .25]	[.15, .28]	[.03, .17]	[.11, .24]	[.08, .21]	[-.04, .10]	[.06, .19]	[-.10, .04]	[-.12, .02]	[-.14, -.00]	[-.18, -.05]	[-.23, -.10]	[-.19, -.05]	[-.13, .00]	[-.24, -.11]	[.18, .31]	[.14, .27]	[.24, .36]	[.06, .20]	[.18, .31]	[.15, .28]	[.14, .27]	[.11, .25]	[.04, .18]	[.16, .29]			
34. DEP5	0.49	0.68	.16**	.21**	.24**	.19**	.27**	.18**	.21**	.22**	.26**	.17**	.23**	.22**	.13**	.17**	-0.04	-0.06	-.08*	-.10**	-.16**	-.19**	-.12**	-.17**	.27**	.32**	.33**	.31**	.30**	.34**	.29**	.27**	.33**	.32**	.24**		
			[.09, .22]	[.14, .27]	[.18, .31]	[.13, .26]	[.20, .33]	[.11, .24]	[.14, .27]	[.15, .28]	[.20, .32]	[.10, .23]	[.17, .30]	[.15, .28]	[.06, .19]	[.10, .23]	[-.11, .03]	[-.13, .01]	[-.14, -.01]	[-.17, -.03]	[-.23, -.09]	[-.25, -.12]	[-.18, -.05]	[-.24, -.10]	[.20, .33]	[.26, .38]	[.27, .39]	[.24, .37]	[.23, .36]	[.28, .40]	[.23, .35]	[.21, .33]	[.26, .39]	[.26, .38]	[.17, .30]		
35. DEP6	0.48	0.71	.19**	.16**	.26**	.21**	.28**	.22**	.23**	.18**	.24**	.20**	.23**	.25**	.09*	.18**	-0.01	-0.05	-.11**	-.15**	-.21**	-.24**	-.16**	-.10**	.28**	.28**	.30**	.30**	.21**	.22**	.22**	.40**	.40**	.37**	.19**	.32**	
			[.13, .26]	[.09, .22]	[.20, .33]	[.14, .27]	[.21, .34]	[.16, .29]	[.16, .29]	[.12, .25]	[.18, .30]	[.13, .26]	[.17, .30]	[.18, .31]	[.02, .15]	[.11, .24]	[-.07, .06]	[-.12, .02]	[-.18, -.05]	[-.21, -.08]	[-.28, -.14]	[-.30, -.17]	[-.23, -.10]	[-.16, -.03]	[.21, .34]	[.21, .34]	[.24, .36]	[.23, .36]	[.15, .28]	[.15, .28]	[.15, .28]	[.34, .46]	[.34, .46]	[.31, .43]	[.12, .25]	[.26, .38]	
36. DEP7	0.45	0.66	.15**	.13**	.18**	.09*	.12**	.13**	.15**	.13**	.18**	0.03	.14**	.14**	0.07	.10**	-0.05	-.10**	-.11**	-.13**	-.08*	-.17**	-.19**	-.20**	.23**	.22**	.18**	.33**	.23**	.19**	.25**	.28**	.31**	.29**	.08*	.25**	.29**
			[.08, .22]	[.06, .20]	[.12, .25]	[.02, .16]	[.05, .18]	[.06, .20]	[.08, .21]	[.06, .20]	[.11, .25]	[-.04, .10]	[.08, .21]	[.07, .20]	[-.00, .14]	[.03, .16]	[-.12, .02]	[-.17, -.03]	[-.17, -.04]	[-.20, -.07]	[-.15, -.01]	[-.23, -.10]	[-.26, -.13]	[-.26, -.13]	[.17, .30]	[.15, .28]	[.11, .25]	[.27, .39]	[.17, .30]	[.12, .26]	[.19, .32]	[.22, .34]	[.24, .37]	[.23, .36]	[.01, .14]	[.18, .31]	[.23, .35]
Note. M and *SD* are used to represent mean and standard deviation, respectively. Values in square brackets indicate the 95% confidence interval for each correlation. The confidence interval is a plausible range of population correlations that could have caused the sample correlation (Cumming, 2014). * indicates *p* < .05. ** indicates *p* < .01.																																					

© 2020 Fischer R et al. *JAMA Network Open.*
